# Wool–Poly(lactic acid) Biocomposite Mats as Degradable Sulphur Carriers: Effects on Forage Elemental Composition and Material Degradation

**DOI:** 10.3390/molecules31203777

**Published:** 2026-10-21

**Authors:** Jakub Barwinek, Zuzanna Flis, Andrzej Czulak, Katarzyna Pamin, Bogna Napruszewska, Edyta Molik, Marcin Niemiec, Piotr Szatkowski

**Affiliations:** 1 Department of Glass Technology and Amorphous Coatings, Faculty of Materials Science and Ceramics, AGH University of Krakow, Al. Mickiewicza 30, 30-059 Krakow, Poland; barwinek@agh.edu.pl; 2 Department of Animal Biotechnology, Faculty of Animal Sciences, University of Agriculture in Krakow, A. Mickiewicz 24/28 Av., 30-059 Krakow, Poland; zuzanna.flis@urk.edu.pl (Z.F.); edyta.molik@urk.edu.pl (E.M.); 3 Łukasiewicz Research Network—Institute of Ceramics and Building Materials, 31-983 Krakow, Poland; andrzej.czulak@icimb.lukasiewicz.gov.pl; 4 Jerzy Haber Institute of Catalysis and Surface Chemistry, Polish Academy of Sciences, Niezapominajek 8, 30-239 Krakow, Poland; katarzyna.pamin@ikifp.edu.pl (K.P.); bogna.napruszewska@ikifp.edu.pl (B.N.); 5 Department of Agricultural and Environmental Chemistry, Faculty of Agriculture and Economics, University of Agriculture in Krakow, 31-120 Kraków, Poland; marcin.niemiec@urk.edu.pl

**Keywords:** sheep wool, keratin, poly(lactic acid), biocomposite mats, sulphur, nutrient release, composting, ICP-OES, dynamic mechanical analysis, circular economy

## Abstract

Low-grade sheep wool is an underused, keratin-rich by-product whose cystine-derived sulphur could be returned to agricultural soils. Carded wool was bonded with sprayed poly(lactic acid) (PLA) hot-melt to obtain porous wool–PLA biocomposite mats, which were exposed in field soil, in a pasture-grass composter and under soil-free outdoor conditions. The elemental composition of the wool, soil, forage, composting material and exposed mats was determined by ICP-OES after 60 days of exposure; each reported value is the mean of at least three analytical determinations, whereas the field layout comprised one plot per treatment. Forage from the plot with buried mats contained 47.8% more S and 21.9% more K than forage from the untreated plot, whereas Ca, Mg, B and Mo concentrations were lower; because the plots were not replicated, these differences are reported as preliminary observations rather than as demonstrated treatment effects. After composting, the mats retained only 17–23% of their initial storage modulus between −30 and 50 °C, while the relaxation of the PLA phase shifted by 7–10 °C towards higher temperatures. The water contact angle decreased from 72.3 ± 10.0° to 55.7 ± 12.1° (six droplets on one mat per condition), while the stylus roughness did not change appreciably. Digital microscopy revealed swelling, fissuring, cavities and fibre exposure along wool–PLA interfaces. A coupled mechanism of PLA ester hydrolysis and keratinolytic release of sulphur is proposed as a working hypothesis that is consistent with, but not yet proven by, the present data. The results support wool–PLA mats as temporary, degradable carriers of keratin-bound sulphur; replicated field trials with yield, nutrient uptake and soil measurements, together with direct degradation markers, are required before any fertilising or soil-improving effect can be claimed.

## 1. Introduction

Sheep wool is one of the oldest natural fibres used by humankind, yet a substantial share of the wool produced worldwide is never processed into textiles and is instead discarded as an agricultural by-product [1,2]. Global raw wool production is estimated at 1.7–2.5 million tonnes per year, of which only around 60% is directed toward the textile industry, leaving hundreds of thousands of tonnes of coarse, contaminated or low-grade wool without an economically viable outlet [1,2,3]. When keratin-rich wastes from livestock production (wool, feathers, hooves, horns and bristles) are considered collectively, the total volume approaches 12 million tonnes annually [1,4]. In the European Union, low-grade wool that is not used for textile purposes is classified as a Category 3 animal by-product under Regulation (EC) No 1069/2009, which restricts its disposal to incineration, composting or landfilling after appropriate processing and imposes a direct economic and logistical burden on sheep farmers [5]. This pressure, combined with the environmental cost of landfilling a slowly biodegradable material, has intensified interest in value-added applications of wool waste that are consistent with circular-economy and bioeconomy principles [3,6,7].

The potential agricultural value of wool originates from the molecular architecture of keratin. Wool fibres consist predominantly of α-keratin, a fibrous protein with a hierarchical α-helical coiled-coil structure stabilised by hydrogen bonds, hydrophobic interactions and a dense network of disulphide crosslinks formed by cystine residues [4,8,9]. Cystine accounts for most of the sulphur in wool, which typically contains approximately 3–4% S on a dry-mass basis, together with 15–16% N and smaller quantities of mineral elements that may become available during progressive decomposition [9,10,11,12]. The compact, crosslinked structure renders keratin largely insoluble in water and resistant to common proteases, which explains both the durability of wool and its slow decomposition in the environment [4,9,13,14]. In soil and compost, keratin breakdown depends on specialised keratinolytic microorganisms that combine disulphide reduction (sulfitolysis) with proteolytic cleavage of the exposed polypeptide chains; the released sulphur-containing amino acids and peptides are subsequently mineralised to sulphate, and organic nitrogen to ammonium and nitrate [13,14,15,16]. Untreated wool should therefore be regarded as a slowly decomposing organic nutrient reservoir rather than as a readily soluble fertiliser.

Several approaches have been investigated to increase the agricultural usability of keratin-rich residues. Alkaline, acid, enzymatic, thermal and microbial treatments convert insoluble keratin into hydrolysates containing amino acids and peptides, and such products have been evaluated as liquid fertilisers and plant-growth-promoting formulations [4,5,16,17,18,19,20,21]. Positive effects of wool- and feather-derived hydrolysates on nutrient use, plant development and soil properties have been reported, although they depend strongly on the hydrolysis route, composition, dose, crop and soil conditions [18,19,20,21]. Intact wool has also been studied as a component of growing media, soil amendments and pelletised organic formulations; its fibrous and hygroscopic structure modifies the physical properties of the surrounding medium, while gradual decomposition supplies organic matter, N, S and other nutrients over time [7,22,23,24,25,26]. The practical performance of untreated wool nevertheless depends on its form, spatial distribution, moisture conditions, microbial accessibility and ability to remain in place during application. Converting carded wool into a coherent mat facilitates handling and placement, but the binder used to stabilise the fibrous network must not itself persist in the environment.

Poly(lactic acid) (PLA) is a bio-based aliphatic polyester that can provide such temporary bonding. Its degradation is initiated by hydrolysis of the backbone ester bonds, which generates carboxyl- and hydroxyl-terminated chains; the carboxylic end groups catalyse further hydrolysis, and the process proceeds preferentially in the amorphous regions of the polymer. Once the molar mass has decreased sufficiently, the resulting oligomers and lactic acid are assimilated by microorganisms and mineralised to CO_2_ and water, particularly under the moist and warm conditions of composting [27,28,29]. A wool–PLA mat therefore combines two chemically distinct degradable phases: a polyester binder that loses cohesion through abiotic and biotic hydrolysis, and a disulphide-crosslinked protein that releases sulphur and nitrogen through keratinolysis. How these processes interact under realistic agricultural conditions, and whether they translate into measurable changes in the elemental composition of the surrounding soil–plant system, has not yet been established.

Although wool hydrolysates are sometimes discussed within the broader field of plant biostimulants [30,31,32,33,34,35], no biostimulant effect is claimed in the present work: under Regulation (EU) 2019/1009, such a claim would require dedicated measurements of plant growth, nutrient-use efficiency, stress tolerance or crop quality [30], which were not performed here.

Despite increasing interest in wool valorisation, little information is available on bonded wool mats designed for direct placement in soil or compost. Earlier studies have primarily addressed wool chemistry, keratin hydrolysis, loose-fibre amendments or pelletised formulations [4,16,17,18,19,20,21,22,23,24], whereas few have combined elemental analysis of the surrounding agricultural matrices with an assessment of the thermomechanical, surface and morphological changes occurring in the material itself. Accordingly, the central research question of this study was whether a carded wool web bonded with a sprayed PLA hot-melt loses its structural integrity under agricultural exposure conditions quickly enough to act as a temporary carrier, while the keratin phase remains a potential source of sulphur for the soil–plant system. The specific objectives were to establish, in a preliminary single-season experiment, (i) whether differences in the elemental profile of forage, soil and composting material can be detected in the presence of the mats and (ii) to what extent 60 days of composting degrade the thermomechanical, surface and morphological integrity of the material. ICP-OES, dynamic mechanical analysis (DMA), contact-angle measurements, stylus profilometry and digital microscopy were the tools used to address these questions. We hypothesised that the PLA bonding phase deteriorates by ester hydrolysis and that the keratin phase releases sulphur through microbial keratinolysis; this coupled mechanism is treated throughout as a working hypothesis to be tested, not as an established conclusion. Because the field plots were not replicated, the agronomic observations are explicitly regarded as preliminary, whereas all reported analytical values are means of at least three determinations.

## 2. Results and Discussion

### 2.1. Elemental Composition of Wool and of the Soil–Plant and Composting Matrices

The concentrations of 29 elements determined in the washed wool, the control soil (G0), forage from the untreated plot (G0) and from the plot with buried mats (G1), the composting material (G2) and the mat exposed under soil-free outdoor conditions for 60 days (G3) are summarised in Table 1. Each value in Table 1 is the mean of at least three analytical determinations (*n* ≥ 3) of the same sample. Because the field design comprised a single plot per treatment and one composter, the between-group differences described below reflect the comparison of single experimental units; they are therefore interpreted as preliminary observations rather than as statistically tested treatment effects. Values that failed plausibility checks are flagged in the table and are not interpreted.

The washed wool contained substantial amounts of Ca (6612 mg kg^−1^), S (5289 mg kg^−1^), Fe (737 mg kg^−1^), Mg (714 mg kg^−1^), Na (589 mg kg^−1^), K (352 mg kg^−1^), Si (252 mg kg^−1^) and P (214 mg kg^−1^), while among the agronomically relevant trace elements Zn (76.9 mg kg^−1^) dominated over Mn, Cu, B and Ni. The comparatively high sulphur content reflects the cystine-rich keratin matrix and agrees with the general characterisation of wool as an organic source of N and S accompanied by smaller amounts of K, P, Mg, Ca, Fe, Cu, Mn and Zn [11,12,25]. The measured value (0.53% S) is, however, well below the 3–4% typically reported for wool keratin [8,9]. Because disulphide-bound sulphur is not readily released from the highly crosslinked fibre, and because the wool was mineralised with the procedure intended for plant material (Section 3.4), this discrepancy most probably indicates incomplete recovery of keratin-bound sulphur during digestion; the absolute wool S value should therefore be considered a lower estimate, and the same reservation applies to the other elements determined in the wool matrix. Re-determination after a dedicated keratin digestion, with ICP-MS confirmation of the trace elements, is in progress.

The control soil was dominated by Ca (47,024 mg kg^−1^), Al (18,793 mg kg^−1^), Fe (14,070 mg kg^−1^), Mg (10,806 mg kg^−1^) and K (5182 mg kg^−1^), whereas its sulphur concentration was only 334 mg kg^−1^. Even with the conservative wool S value, the mats thus represented a sulphur reservoir at least an order of magnitude more concentrated than the surrounding soil, while most of the Al, Fe, Mg, Ca, Ba, Sr and Li in the system originated from the mineral soil matrix rather than from the wool. Increases in soil- or compost-associated Al and Fe cannot, therefore, be attributed to keratin degradation.

**Figure 1:**
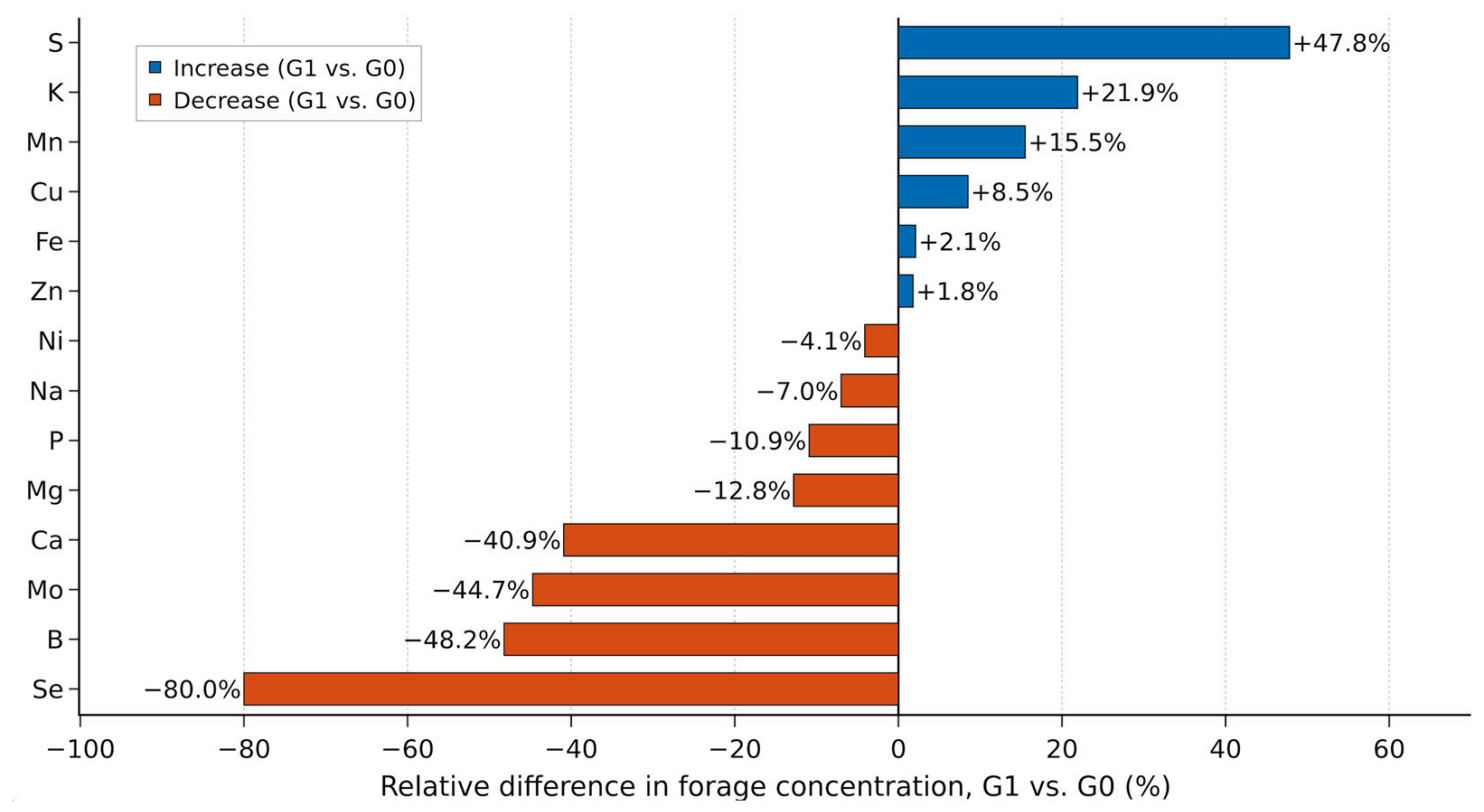
Relative difference in the concentration of macro- and micronutrients in forage from the plot with buried wool–PLA mats (G1) compared with forage from the untreated control plot (G0). Values calculated from the single determinations listed in Table 1.

The most informative comparison for evaluating the agronomic effect of the mats is between forage from the untreated plot (G0) and forage from the plot containing the buried mats (G1), because both samples represent the same biological matrix (Figure 1). Relative to G0, G1 forage contained 47.8% more S (2997 vs. 2028 mg kg^−1^), 21.9% more K (28,528 vs. 23,402 mg kg^−1^), 15.5% more Mn, 8.5% more Cu, 2.1% more Fe and 1.8% more Zn. The sulphur response was the clearest treatment-associated change and is mechanistically consistent with the gradual microbial breakdown of sulphur-rich keratin: reduction in the cystine disulphide bonds and proteolytic cleavage release cysteine- and methionine-containing peptides and amino acids, which are mineralised to sulphate that enters the soil solution and becomes available for root uptake [13,14,15] (see Section 2.5). It must be stressed, however, that G0 and G1 are single plots, so that spatial heterogeneity of the soil, sward composition and sampling position cannot be separated from the effect of the mats; the same reservation applies, symmetrically, to the decreases discussed below. Aluminium (+17.0%) and Li (+6.7%) were also higher in G1 forage; as neither element is a plant nutrient and both are abundant in the soil, the Al concentrations of 370–440 mg kg^−1^ most likely indicate adhering soil dust on the harvested biomass. When the forage concentrations are normalised to Al to correct for this contamination, the S/Al ratio remains higher in G1 than in G0 (6.9 vs. 5.4, i.e., +26%), whereas the K/Al ratio differs by only +4% (65.4 vs. 62.8). The sulphur difference is thus not explained by soil contamination alone, while most of the apparent potassium difference disappears after normalisation. The Cr (36–40 mg kg^−1^) and Ni (22–23 mg kg^−1^) concentrations in forage are high for pasture grass, and Cr exceeds the value found in the soil; since both elements were virtually identical in G0, G1 and the composting material, a contamination or spectral-interference effect common to all plant-type digests is suspected, and these two elements are not interpreted.

The concurrent increase in forage K may indicate improved potassium availability or uptake in the presence of the mats, although, as noted above, most of this difference vanishes after normalisation to Al and it should therefore be regarded with caution. Uncomposted wool has previously been shown to act as a slow-release source of N, S, P and K and to increase plant K concentrations [25,26]. In the present experiment, however, the washed wool contained only 352 mg kg^−1^ K, so the 21.9% increase cannot be assigned solely to direct release from the fibres. An indirect mechanism is plausible: the fibrous, hygroscopic mat may have modified moisture retention and rhizosphere conditions, facilitating root activity and mobilisation of the potassium already present in the soil, in line with reports that wool amendments improve soil water retention, biological activity and nutrient availability [23,25]. The modest increases in Mn, Cu, Fe and Zn are consistent with a small directly released fraction or with changes in micronutrient solubility caused by organic compounds formed during keratin degradation, but the 2.1% and 1.8% differences for Fe and Zn lie within the range expected from sampling and analytical variability.

Not all nutrients increased in G1 forage. Compared with G0, the concentrations of B, Ca, Mg, P, Mo and Sr were lower by 48.2, 40.9, 12.8, 10.9, 44.7 and 50.2%, respectively (Se was close to the limit of quantification in both samples and is not interpreted). Tissue concentration reflects the balance between nutrient acquisition and dry-matter accumulation, and enhanced growth can dilute an absorbed element over a larger biomass; concentration data should therefore ideally be interpreted together with dry-matter yield and total uptake per plot. Differences in sward composition, developmental stage, soil heterogeneity and sampling position relative to the mat may also have contributed. The same sources of uncertainty that could produce a 50% decrease could equally produce the observed increases; an asymmetric interpretation is therefore avoided, and both directions of change are reported as observations from unreplicated plots. Dry-matter yield, total nutrient uptake and plant-available soil nutrients were not determined in this preliminary season and are included in the replicated trial (Section 2.7). The simultaneous decrease in Ca and Mg and increase in K is consistent with cation competition at root and exchange sites, where greater K uptake can coincide with lower tissue Ca and Mg concentrations without a decline in their total uptake. Similar wool-fertilisation studies have reported increased plant K alongside lower Ca or B concentrations [25,26]. The G1 pattern is therefore best described as a redistribution of the forage mineral profile, with pronounced enrichment in S and K, rather than as a uniform increase in all nutrients.

The G2 composting material exhibited the highest concentrations of several nutrients. Relative to G0 forage, it contained 66.6% more S, 65.4% more Mg, 28.8% more Ca, 17.3% more P, 164.4% more Mn, 94.6% more Zn and 51.6% more Cu. Composting provides sustained moisture, intimate contact between the keratinous substrate and decomposing plant biomass, and a dense microbial community, all of which favour keratinolysis and nutrient redistribution [7,15]. At the same time, G2 contained markedly more Al (4218 mg kg^−1^), Fe (2862 mg kg^−1^), Co, Li, Ba, Na and Pb (10.3 mg kg^−1^) than G0 forage. These elements are unlikely to originate from the wool, whose Al, Fe and Pb concentrations were 406, 737 and 2.1 mg kg^−1^, respectively; the profile instead indicates the presence of adhering soil or another mineral fraction, and the Pb concentration in G2 was indeed close to that of the control soil (9.6 mg kg^−1^). G2 therefore represents a composting material whose composition reflects the combined effects of wool degradation, grass decomposition, dry-mass loss and mineral contamination, and it should not be interpreted as plant uptake. Potassium was the exception to the general enrichment: its concentration in G2 (3277 mg kg^−1^) was 86.0% lower than in G0 forage. Because K occurs predominantly as the mobile K^+^ ion rather than as a structural component of organic matter, leaching during moist composting is a plausible explanation, which could be verified by analysis of the compost leachate.

The G3 mat exposed for 60 days under soil-free outdoor conditions differed strongly from the washed wool, but its data are not used for mechanistic interpretation. Its apparent K content (60,306 mg kg^−1^, approximately 171-fold that of the washed wool) and Na content (3753 mg kg^−1^) cannot be reconciled with any plausible process: degradation cannot create elements, and a 171-fold enrichment would require the loss of more than 99% of the non-potassium mass of a still recoverable mat. These values are therefore flagged in Table 1 as unreliable. For consistency, the S concentration of the G3 mat (32,401 mg kg^−1^, 3.24%) is treated with the same reservation: although it falls within the 3–4% range reported for wool keratin [8,9], it was obtained from the same digest and cannot be used as evidence either of sulphur release (a mat that releases S should lose it, and G3 had no contact with soil microflora) or of the true S content of the wool. The retained G3 material will be re-analysed by ICP-MS after a dedicated keratin digestion.

A similar caution applies to the elevated As (187 mg kg^−1^), Ga, Bi and Be concentrations obtained for the washed wool, which were determined only in this matrix. For comparison, Regulation (EU) 2019/1009 sets a limit of 40 mg kg^−1^ dry matter for inorganic As in organic fertilisers and organic soil improvers [30]. The ICP-OES value represents total rather than speciated As and cannot be compared directly with this threshold; moreover, As determination by ICP-OES in Ca- and Fe-rich digests is susceptible to spectral interferences. The value was not confirmed by an independent technique such as ICP-MS or hydride-generation atomic spectrometry; together with Ga, Bi and Be, it is flagged as unreliable in Table 1, excluded from interpretation and scheduled for re-determination by ICP-MS. No agronomic safety claim is made on its basis. Overall, the clearest observation of an interaction between the mats and the soil–plant system is the forage-to-forage comparison between G1 and G0, which showed a pronounced difference in S that persisted after normalisation to Al, whereas composting promoted broader nutrient accumulation in the composting material. Given the unreplicated design, these observations define hypotheses for the replicated trial rather than demonstrated effects.

### 2.2. Thermomechanical Behaviour of the Wool–PLA Mats

**Figure 2:**
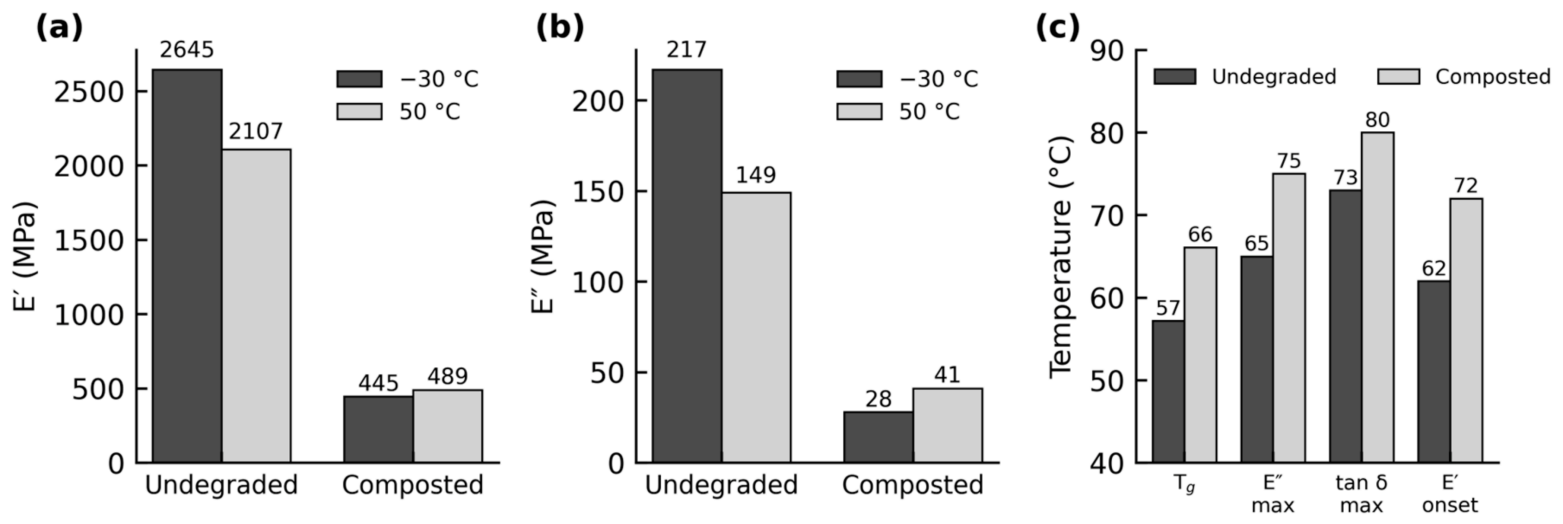
(**a**) Storage modulus and (**b**) loss modulus of the wool–PLA biocomposite mat at −30 and 50 °C, and (**c**) glass-transition descriptors before and after composting.

**Figure 3:**
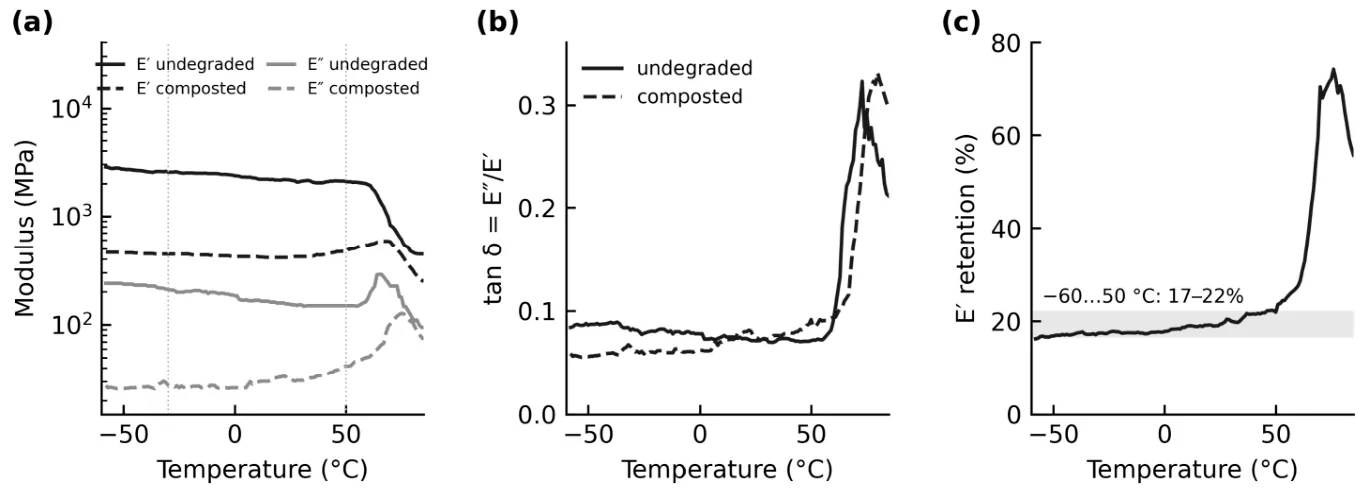
(**a**) Storage and loss modulus, (**b**) damping factor (tan δ) and (**c**) retention of storage modulus (E′_composted_/E′_undegraded_) as functions of temperature. Dotted vertical lines in (**a**) indicate −30 and 50 °C.

DMA revealed a pronounced deterioration in the load-bearing capacity of the wool–PLA mats after composting (Table 2, Figure 2 and Figure 3). The values in Table 2 are means of three specimens per condition (*n* = 3) cut from the same mat; representative curves of one specimen are shown in Figure 2 and Figure 3. At −30 °C, the storage modulus (E′) decreased from 2645 MPa for the undegraded material to 445 MPa after composting, corresponding to an 83% reduction. At 25 °C, E′ decreased from approximately 2160 to 420 MPa (81%), and at 50 °C from 2107 to 489 MPa (77%). The composted material thus retained only 17–23% of its original storage modulus in the service-temperature range. The nearly constant retention level between −60 and 50 °C (Figure 3c) shows that the reduction was not confined to the vicinity of the glass transition but reflected an extensive loss of structural continuity and stress-transfer efficiency throughout the investigated interval. The loss modulus (E″) was also markedly reduced, from 217 to 28 MPa at −30 °C (87%) and from 149 to 41 MPa at 50 °C (72%) (Figure 2b), demonstrating that composting decreased not only the elastic response but also the energy dissipated through the original polymer–fibre structure. Hydrolytic chain scission of PLA under composting conditions is known to cause a rapid loss of molar mass and mechanical properties [27,28,29]; in the present material, the effect is additionally attributable to weakened PLA bonding points, deterioration of the wool–PLA interface, fibre rearrangement and the formation of discontinuities. The absolute E′ values of 2.1–2.6 GPa for the undegraded mat require comment. They are of the order of bulk PLA rather than of a loose non-woven, because the sprayed PLA formed a continuous, partly consolidated skin on the surface of the web and the modulus was calculated from the nominal external cross-section of the specimen. The reported values are therefore apparent moduli of the bonded structure, not intrinsic moduli of the wool–PLA network, and they should be compared only between the two conditions tested here. After composting, the cross-section additionally contained soil, water and voids, so that the apparent modulus also reflects the altered geometry; this is a further reason to regard the retention values as descriptive.

The temperature dependence of the dynamic response also changed after composting (Figure 3a,b). The undegraded mat exhibited the expected gradual reduction in E′ on heating, by approximately 20% between −30 and 50 °C. In contrast, the composted material showed a modest increase in E′ from 445 to 489 MPa over the same interval and an increase of approximately 30% between 40 and 68 °C, immediately before the main relaxation. Simultaneously, E″ of the composted specimen rose progressively from approximately 10 °C and formed a broad shoulder below the principal relaxation, so that tan δ was lower after composting below approximately 15 °C but higher between approximately 20 and 60 °C. This atypical pre-transition stiffening may be related to the release of moisture absorbed by the hygroscopic wool and the partly hydrolysed PLA phase: water initially acts as a plasticiser, whereas its removal during heating reduces segmental mobility and temporarily increases the apparent stiffness. Physical ageing of the amorphous PLA fraction may also contribute. Because the water content was not monitored immediately before and after DMA, this interpretation remains a hypothesis that requires confirmation by gravimetric, thermogravimetric or sorption measurements. The full temperature sweeps of E′ and E″ are provided in Figure S1 as a Supplementary Material.

All glass-transition descriptors shifted towards higher temperatures after composting (Table 2, Figure 2c): T_g_ and the E′ decrease from approximately 62 to 72 °C. These consistent shifts of 7–10 °C occurred despite the pronounced decrease in absolute modulus. Hydrolysis of PLA preferentially affects the more accessible amorphous regions, and the shortened chains produced by scission can undergo secondary crystallisation, increasing the relative proportion of ordered domains [27,28]. Such degradation-induced rearrangement constrains the remaining amorphous phase and shifts its relaxation to higher temperatures; confirmation of this mechanism in the present biocomposite would require correlation with DSC-derived crystallinity and molar-mass measurements. The unchanged height of the tan δ maximum (approximately 0.32–0.33) indicates that the principal PLA relaxation remained detectable after composting. The higher transition temperatures therefore do not signify improved mechanical performance: the marked reductions in E′ and E″ demonstrate that composting severely compromised macroscopic integrity, whereas the surviving polymer-rich regions underwent a shift in their relaxation behaviour. Because lower amplitude and force settings were required for the composted material, and E′ of a non-linear fibrous structure depends on strain amplitude, the 17–23% retention is a semi-quantitative comparison; the magnitude of the modulus loss (about 80%) is, however, far larger than the amplitude dependence expected within the near-linear range and provides strong descriptive evidence of degradation. The moisture content of the specimens was not determined immediately before testing, and alternative explanations of the same DMA result—swelling and delamination of the wool, soil ingress or mechanical damage during exposure—cannot be excluded without complementary mass-loss, SEC and DSC data. The interpretation of the T_g_ shift as degradation-induced secondary crystallisation is therefore offered as a hypothesis, and replicate measurements on independent mats are needed to establish material-wide variability.

### 2.3. Surface Wettability and Roughness

**Figure 4:**
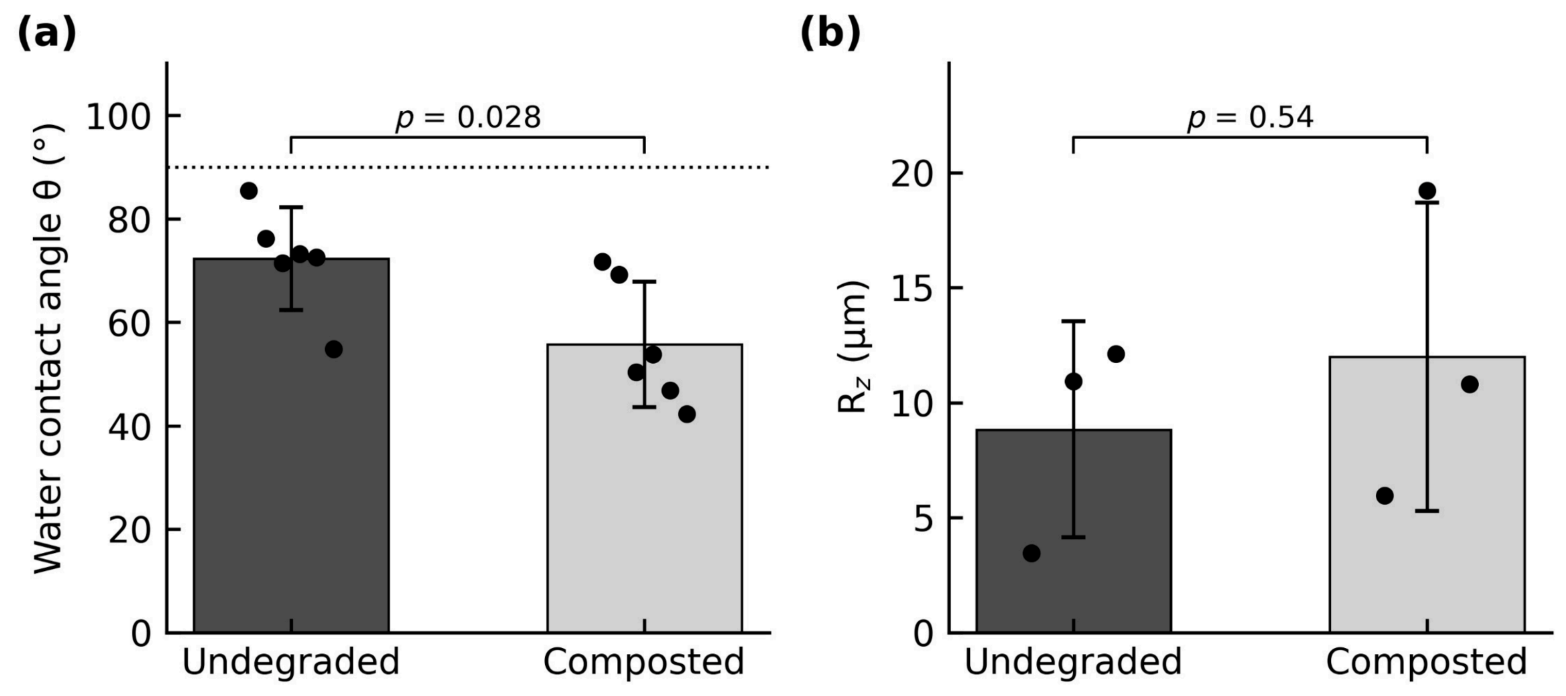
(**a**) Static water contact angle and (**b**) maximum height of the roughness profile (*R*_z_) of the wool–PLA mats before and after composting. Bars represent mean ± SD; points show individual measurements; the dotted line indicates θ = 90°.

Composting markedly increased the wettability of the wool–PLA mats (Table 3, Figure 4a). The mean water contact angle decreased from 72.3 ± 10.0° for the undegraded material to 55.7 ± 12.1° after composting, a reduction of 16.5° (approximately 23%) with a 95% confidence interval of −30.8° to −2.2°. Welch’s *t*-test (*p* = 0.028), the Mann–Whitney U test (*p* = 0.015) and Hedges’ g = −1.38 describe the separation between the two sets of droplets; because all six droplets per condition were deposited on a single mat, they are technical rather than independent replicates, and these statistics characterise the within-mat difference only and do not constitute a test of a treatment effect at the material level. Five of the six contact angles measured on the undegraded mat exceeded 70°, whereas four of the six measured after composting were below 55°. Some droplets were immediately absorbed by exposed fibres and could not be measured; because these were excluded, the reported angles are conservative estimates and the effective wettability of the porous surface was likely greater. It is acknowledged that a static contact angle on a porous, fibrous and soil-contaminated surface is an apparent value governed by partial imbibition and mixed wetting regimes; it is used here only as a relative indicator of surface polarity. The lower contact angle is consistent with the chemistry of PLA hydrolysis: cleavage of ester bonds produces hydroxyl- and carboxyl-terminated chains, increasing the density of polar groups at the surface [28]. Partial weakening of the sprayed PLA bonding points may additionally have exposed a greater proportion of hygroscopic wool fibres bearing polar keratin side groups, and adsorption of organic compounds and microbial residues from the compost may also have contributed; these mechanisms require confirmation by surface-sensitive chemical analysis such as ATR-FTIR or XPS.

In contrast, the stylus roughness parameter *R*_z_ did not change appreciably (Figure 4b). The mean *R*_z_ + 16.9 µm; Welch’s *t*-test *p* = 0.54; Mann–Whitney *p* = 1.00; Hedges’ g = 0.44). Given the large dispersion and small number of profiles, moderate local roughening cannot be excluded, but there is no evidence of extensive surface erosion. It is also acknowledged that stylus profilometry with a 0.8 mm cut-off characterises micrometre-scale texture only, whereas the digital-microscope reconstructions (Section 2.4) reveal relief of 600–900 µm; the *R*_z_ values therefore describe the local texture of intact PLA-rich regions and not the macroscopic damage of the mats. Areal parameters (S_a_, S_z_) from optical profilometry will be used in future work. The combination of a significant decrease in contact angle and a non-significant change in *R*_z_ indicates that composting altered the chemical and interfacial character of the surface more strongly than its micrometre-scale topography, so the enhanced wettability cannot be attributed primarily to roughness development. Together with the DMA results, this suggests that the dominant degradation processes occurred within the fibrous structure and at the wool–PLA bonding regions rather than through uniform erosion of the external PLA-rich surface. Increased wettability may in turn facilitate moisture ingress and microbial colonisation, although a direct relationship between contact angle and biodegradation rate was not established here.

### 2.4. Morphology of the Exposed Mats

**Figure 5:**
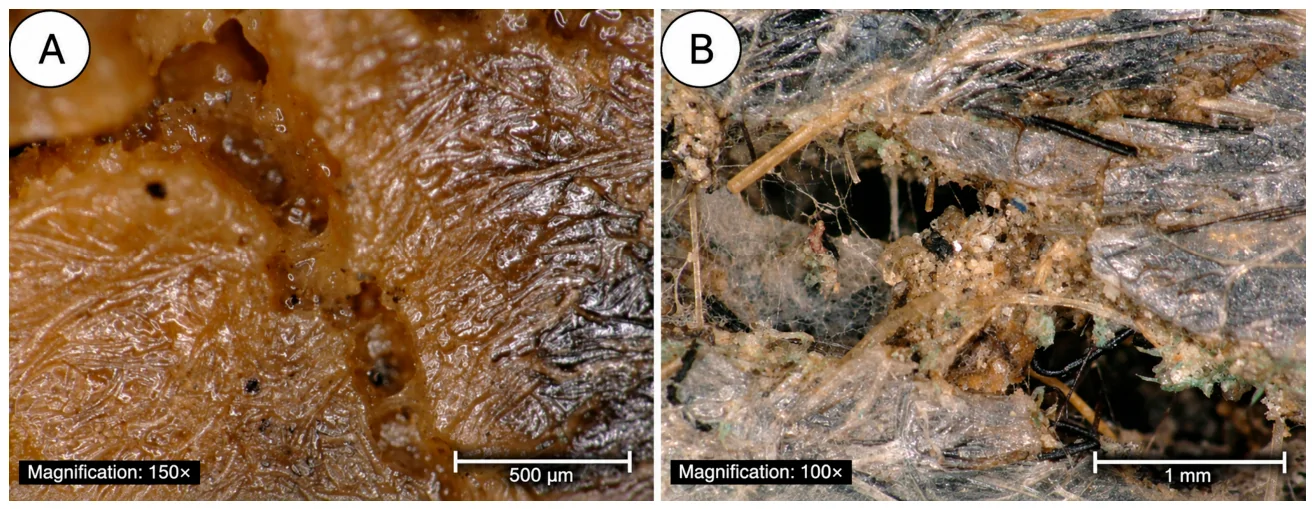
Digital-microscope images of the exposed regions of the wool–PLA mats: (**A**) specimen S1 showing a swollen and fissured surface with the fibre network embedded in a gel-like layer (150×); (**B**) specimen S2 showing cavities bridged by exposed wool fibres, together with retained soil particles and greenish deposits (100×).

**Figure 6:**
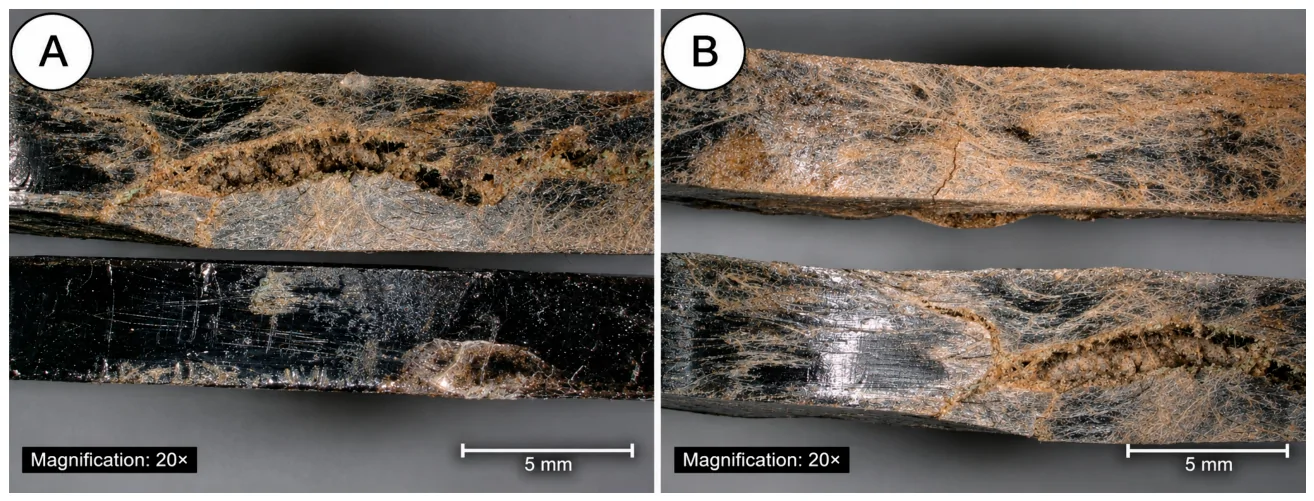
Side views of the exposed regions of the wool–PLA mats: (**A**) upper and lower views of specimen S1; (**B**) upper and lower views of specimen S2 (20×).

**Figure 7:**
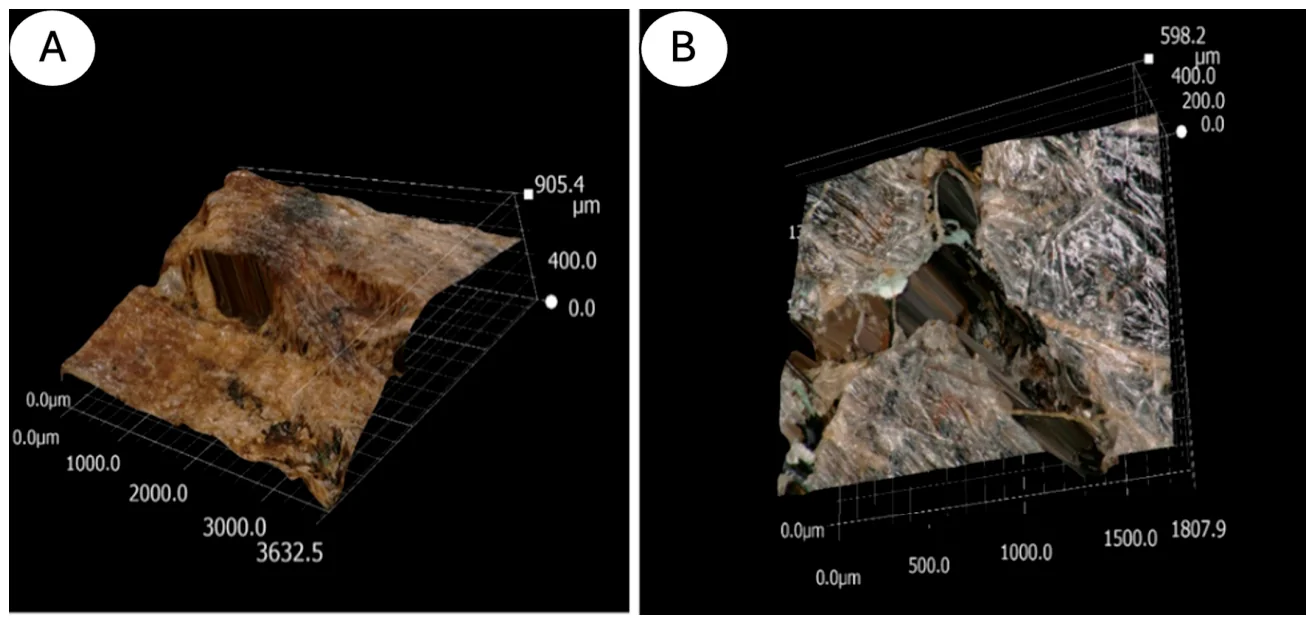
Three-dimensional reconstructions of the exposed surfaces of the wool–PLA mats obtained by depth composition (VHX-5000; Keyence Corporation, Osaka, Japan): (**A**) specimen S1, displayed vertical range approximately 905.4 µm; (**B**) specimen S2, displayed vertical range approximately 598.2 µm. The height ranges include local surface relief, specimen deformation and possible inclination of the analysed regions.

Digital microscopy revealed pronounced and spatially heterogeneous morphological changes in the two mat specimens recovered after 60 days of exposure: S1 was retrieved from the composter (G2) and S2 from the field plot (G1). No dedicated micrograph of an unexposed mat was recorded; the freshly manufactured mats had a smooth, continuous, PLA-rich surface and the changes described below are assessed relative to that initial state. The G3 mats were not examined microscopically. Both exhibited locally preserved PLA-rich regions alongside strongly deteriorated areas containing cracks, cavities, exposed wool fibres and retained soil or compost particles (Figure 5, Figure 6 and Figure 7), indicating that deterioration developed preferentially around structural discontinuities, fibre-rich regions and weakened wool–PLA interfaces. At 150× magnification, specimen S1 showed a swollen, irregular surface with fissures, rounded depressions and locally collapsed regions (Figure 5A). The wool-fibre network remained visible within a brown, gel-like surface layer, indicating hydration and a substantial loss of cohesion in the outer part of the mat; the chemical identity of this layer (biofilm, humic material or partially hydrolysed PLA), however, cannot be established from optical images alone, and SEM–EDS and ATR-FTIR examination is planned. Specimen S2 displayed a more open and fragmented morphology (Figure 5B), with large cavities crossed by individual wool fibres and fibre bundles, and with soil particles and brown or greenish deposits retained in the cavities and on the exposed fibres. The fibre bridges extending across the cavities show that the wool network maintained a degree of local connectivity despite the deterioration of the surrounding PLA-rich regions.

The side views recorded at 20× magnification show that the changes extended beyond superficial discoloration and affected the internal integrity of both mats (Figure 6). Specimen S1 exhibited longitudinal fissures, local separation of adjacent layers and regions of exposed fibrous structure, whereas specimen S2 showed larger openings and more extensive cavities with soil or compost particles retained between separated fragments. In both cases, fissures developed predominantly through fibre-rich regions, indicating that the wool–PLA interfaces and discontinuities in the sprayed bonding phase acted as preferential pathways for moisture penetration. Moisture uptake by the hygroscopic wool fibres may have caused swelling and local stresses within the heterogeneous structure, while concurrent weakening of the PLA bonding points facilitated fibre rearrangement, crack propagation and separation of adjacent regions.

The three-dimensional reconstructions confirmed the pronounced topographical heterogeneity (Figure 7). Specimen S1 exhibited an extensively deformed surface with a deep central depression surrounded by elevated and locally folded regions (displayed vertical range approximately 905 µm over a field approximately 3.63 mm wide), consistent with local swelling, collapse and separation of material around an open cavity. The surface of specimen S2 consisted of irregular fragments separated by cavities and steep height transitions (approximately 598 µm over a field approximately 1.81 mm wide), corresponding to the fibre-bridged cavities visible in Figure 5B. Because the reconstructed fields differed in lateral dimensions and the height ranges may include specimen inclination, curvature and displacement of fragments, the reconstructions are interpreted as qualitative evidence of deformation and loss of surface continuity; the height ranges are not equivalent to the profilometric *R*_z_ values. The apparent discrepancy between the limited change in *R*_z_ and the pronounced local damage observed microscopically results from the different spatial scales of the two techniques: stylus profilometry samples selected profiles, whereas digital microscopy captures macroscopic cavities, cracks and layer separation.

### 2.5. Proposed Deterioration Mechanism (Working Hypothesis) and Implications

**Scheme 1:**
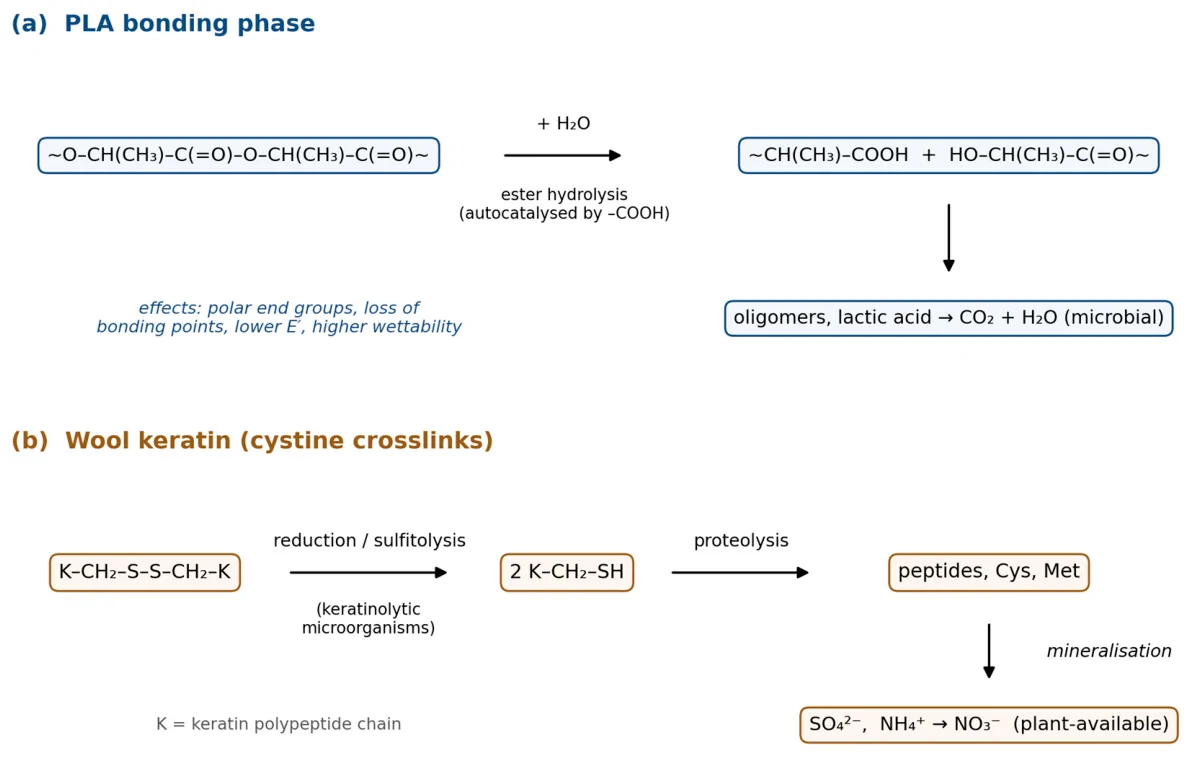
Proposed chemical pathways underlying the deterioration of wool–PLA mats in soil and compost: (**a**) hydrolysis of ester bonds in the PLA bonding phase, autocatalysed by the carboxylic end groups formed, followed by microbial mineralisation of oligomers and lactic acid; (**b**) reductive cleavage of cystine disulphide crosslinks and proteolysis of wool keratin by keratinolytic microorganisms, followed by mineralisation to plant-available sulphate and inorganic nitrogen.

Taken together, the results are compatible with a coupled deterioration mechanism involving the two chemically distinct phases of the mat (Scheme 1), which is put forward as a working hypothesis rather than as a demonstrated pathway. In the PLA bonding phase, water uptake initiates hydrolytic cleavage of the backbone ester bonds, generating carboxyl- and hydroxyl-terminated chains; the acidic end groups catalyse further scission, and the resulting oligomers and lactic acid are subsequently accessible to microbial assimilation [27,28,29]. This sequence is consistent with the loss of storage and loss modulus, the shift of the PLA relaxation to higher temperatures and the increased wettability observed after composting. In the keratin phase, microbial reduction or sulfitolysis of cystine disulphide bonds exposes the polypeptide chains to keratinolytic proteases, releasing sulphur-containing peptides and amino acids that are mineralised to sulphate, while organic nitrogen is converted to ammonium and nitrate [13,14,15,16]. The higher S concentration of forage from the plot with buried mats is consistent with this pathway, but keratin degradation itself was not measured: sulphate, mineral nitrogen, keratinase activity, microbial community composition, CO_2_ evolution and mass loss were not determined, and the composition of the G3 mat is not used as evidence (Section 2.1). The keratin half of the proposed mechanism is therefore inferred from the literature and from the morphological evidence of fibre exposure, not from direct measurements.

The two processes are likely to reinforce each other. Swelling of the hygroscopic wool generates local stresses at the wool–PLA interfaces and promotes water transport into the bonding phase, accelerating ester hydrolysis, while the progressive loss of PLA bonding points opens the fibrous network to moisture and microorganisms, facilitating keratinolysis. From an application perspective, this behaviour is desirable: the mat provides temporary cohesion during handling and placement and then loses its mechanical persistence, while the keratin phase acts as a slowly decomposing reservoir of organically bound sulphur. Direct evidence for the hypothesis will require monitoring of PLA molar mass (SEC) and crystallinity (DSC), surface chemistry (ATR-FTIR, XPS), mass loss, CO_2_ evolution, keratinase activity and the release of sulphate and mineral nitrogen; these measurements form part of the ongoing replicated study.

### 2.6. Safety and Regulatory Considerations

Raw sheep wool is a Category 3 animal by-product under Regulation (EC) No 1069/2009 [5], and its placement in soil or compost is subject to the processing and marketing rules for animal by-products and, where a fertilising product is intended, to Regulation (EU) 2019/1009 [30]. Two groups of risk must be addressed before any practical use. First, untreated wool may carry zoonotic pathogens (e.g., spores of *Bacillus anthracis*, *Coxiella burnetii* or *Salmonella* spp.), ectoparasites and residues of veterinary products such as insecticidal or anthelmintic agents applied to the animals. The mild 30 °C scouring used here removes soluble and particulate contaminants but is not a sanitising treatment; a validated hygienisation step (e.g., composting at ≥70 °C or another approved processing method) would be required for any product placed on the market. Pathogen load and drug residues were not determined in this study, which used wool from a single, veterinary-supervised experimental flock. Second, the PLA binder, although industrially compostable, hydrolyses slowly at field-soil temperatures, so that partially degraded PLA fragments may persist in soil for an extended period. Although PLA is not persistent in the sense of conventional polyolefins and its ultimate hydrolysis product is lactic acid, the formation of bio-based microplastic fragments during field use, their residence time and their effects on soil biota should be quantified (e.g., by mass balance, SEC and respirometry) before the mats can be regarded as fully degradable in situ. Regulation (EU) 2019/1009 also sets contaminant limits for organic soil improvers (e.g., 40 mg kg^−1^ inorganic As, 120 mg kg^−1^ Pb and 50 mg kg^−1^ Ni); the preliminary and partly unverified elemental data for wool presented in Section 2.1 do not permit a compliance statement, and verified ICP-MS data will be required.

### 2.7. Limitations of the Study and Outlook

The present work is a single-season exploratory study, and its limitations are stated explicitly. (i) The field design comprised one plot per treatment and one composter, without wool-only, PLA-only or empty-composter controls, so that between-group differences cannot be separated from spatial heterogeneity and are not statistically testable; the reported means (*n* ≥ 3) describe analytical and within-mat variability only. (ii) Soil physicochemical properties (pH, electrical conductivity, organic matter, water retention, available S and mineral N), dry-matter yield and total nutrient uptake were not measured, and no claim of improved soil fertility is therefore made. (iii) Keratin degradation and PLA hydrolysis were not measured directly, and the proposed coupled mechanism remains a hypothesis. (iv) Several ICP-OES values (As, Be, Bi and Ga in wool; K and Na in the G3 mat; Cr and Ni in all plant-type matrices) are analytically doubtful and are flagged rather than interpreted. (v) Only the day-60 sampling is reported, so the degradation kinetics over the full 123-day season are not described. A replicated trial has been designed on the basis of these results and comprises three randomised plots per treatment, three forage samples per plot with three analytical replicates each, wool-only and PLA-only mats and an empty composter as additional controls, soil pH, EC, organic matter, available S and mineral N, dry-matter yield and total nutrient uptake, monitoring of composter temperature and moisture, and direct degradation markers (mass loss, SEC, DSC, ATR-FTIR, CO_2_ evolution, sulphate, keratinase activity), with sampling at 30, 60, 90 and 123 days.

## 3. Materials and Methods

### 3.1. Materials

The sheep wool was provided by the University of Agriculture in Krakow (Poland) and originated from the Experimental Station of the Department of Animal Biotechnology in Bielany (Kraków district). It comprised mixed fleeces collected as a by-product of routine shearing and was representative of the meat–wool breeds typically raised in Poland. The PLA hot-melt consisted exclusively of PLA granules (NatureWorks LLC, Plymouth, MN, USA; grade 4032D) without auxiliary binders, solvents or processing additives.

**Figure 8:**
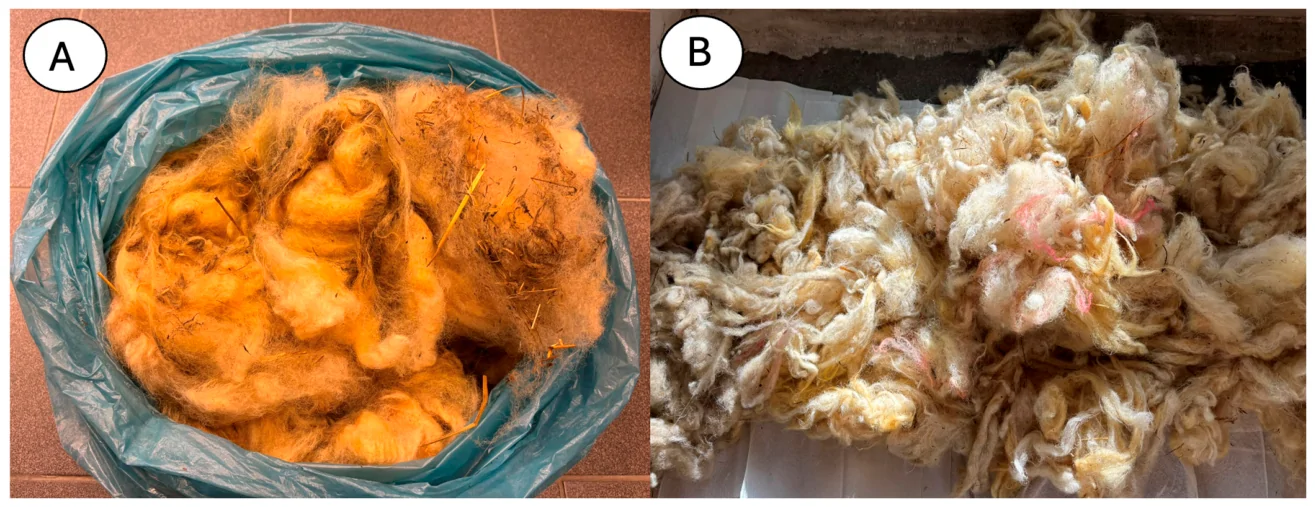
Macroscopic appearance of the sheep wool: (**A**) raw fleece before washing; (**B**) fibre mass after scouring and rinsing.

Before composite manufacture, the fleeces were scoured in a front-loading domestic washing machine. To limit felting and promote water circulation, the raw wool was enclosed in loosely filled mesh laundry bags. Washing was performed at 30 °C with a mild liquid grey soap (Biały Jeleń, Ostrzeszów, Poland) dosed according to the manufacturer’s guidance for delicate textiles; the temperature was not allowed to exceed 30 °C, because at higher temperatures, particularly combined with mechanical agitation, the cuticular scales of the fibres interlock irreversibly and cause felting. The wool was rinsed twice with fresh water to remove soap residues, particulate soiling and water-soluble contaminants, teased apart by hand and air-dried at ambient temperature in a thin, loose layer. Low-temperature drying was chosen to avoid partial thermal denaturation, embrittlement and yellowing of keratin and to preserve the crimp, elasticity and open structure of the fibre mass. The appearance of the wool before and after washing is shown in Figure 8.

### 3.2. Fabrication of the Wool–PLA Mats

**Figure 9:**
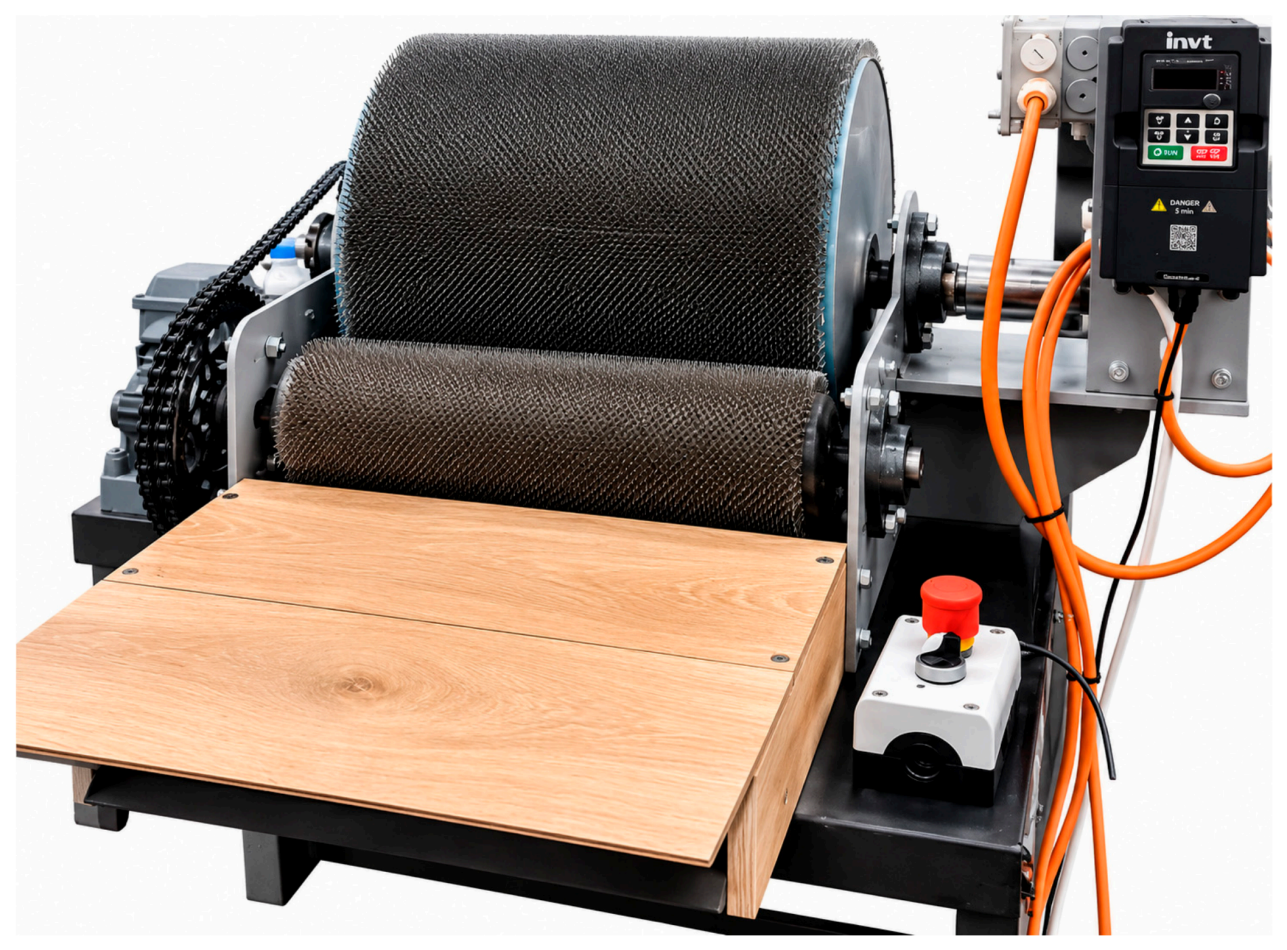
Custom-built laboratory carding device used for mechanical opening and blending of the scoured wool.

The scoured fibres were opened and homogenised on a purpose-built laboratory carding unit consisting of two cylindrical rollers faced with metallic card clothing, mounted on a common frame and linked through a shaft-and-clutch arrangement (Figure 9). The rollers were driven by a stepper motor, allowing the peripheral speed and direction of rotation to be regulated, while an adjustable controller set the carding intensity and fibre dwell time. Small portions of the washed wool were fed by hand into the nip between the counter-rotating rollers and processed repeatedly until an evenly opened, thoroughly blended, flat fibre web was obtained.

**Figure 10:**
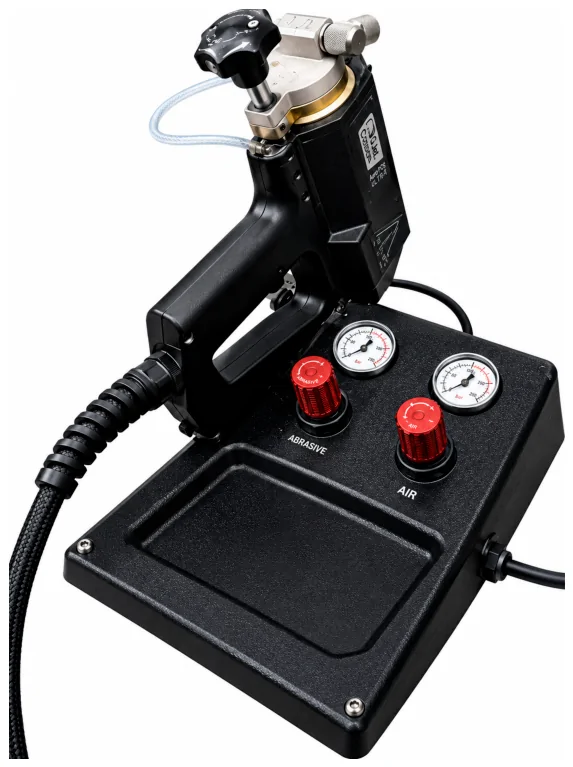
Pneumatic hot-melt spray gun (REKA Klebetechnik, Germany) used to apply the molten PLA to the carded wool web.

**Figure 11:**
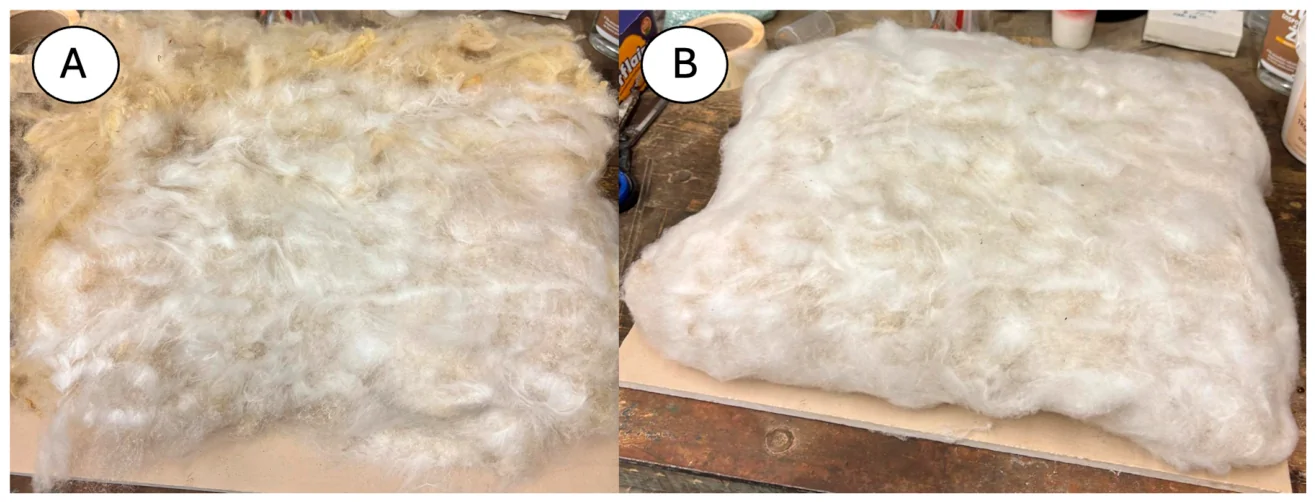
Manufacture of the wool–PLA mats: (**A**) carded wool web during pneumatic application of the molten PLA; (**B**) finished mats after spraying.

The carded web was bonded by spraying the molten PLA directly onto its surface using a pneumatic hot-melt spray gun (REKA Klebetechnik GmbH & Co. KG, Eggenstein-Leopoldshafen, Germany; Figure 10) connected to a piston air compressor that supplied the air required to convey and atomise the melt. The nozzle was heated to 210 °C. The molten polymer formed bonding points between adjacent fibres while preserving the porous structure of the web (Figure 11). No drying stage was required, because hot-melt systems develop cohesion through cooling and resolidification rather than through evaporation of a liquid carrier; once the deposited PLA had cooled to ambient temperature, the mats were ready for use.

### 3.3. Experimental Design and Exposure Conditions

Placed in the composter as a control for binder persistence; its characterisation belongs to a separate study and will be reported together with the replicated trial. No wool-only or PLA-only mats and no composter without a mat were included in this preliminary season. The field and composting trials ran from 20 May to 20 September 2026 (123 days), covering the main vegetation season. The field plots were located at the Experimental Station in Bielany, University of Agriculture in Krakow, ul. Rędzina 1B, 30-274 Krakow, Poland; the total elemental composition of the control topsoil is given in Table 1, whereas soil pH, electrical conductivity, organic matter and plant-available nutrients were not determined in this season. Each plot measured 1.0 × 1.0 m. The mats had an areal density of approximately 750 g m^−2^ and a PLA content of approximately 20 wt%, corresponding to a total mat mass of 150 g per plot; they were laid on the soil surface and covered with approximately 5 cm of soil.

The experimental site was covered by permanent pasture vegetation composed predominantly of grasses. Because no quantitative botanical survey was conducted, the dominant species and their relative proportions were not determined. The composter was a garden-type grass composter with a nominal capacity of approximately 300 L; its temperature, moisture content and pH were not monitored.

After 60 days, samples of soil (G0; 0–20 cm layer, a composite of five individual cores), forage (G0 and G1) and composting material (G2) were collected for elemental analysis. The developmental stage of the pasture vegetation was not formally assessed. In G1, forage was harvested from an area of 0.42 m^2^ comprising the surface directly above the buried 0.40 × 0.50 m mat and a 10 cm wide zone surrounding its edges. An equivalent 0.42 m^2^ area was harvested from the central part of the control plot (G0). The forage samples were dried at 60 °C and milled. The G2 composting material was collected as a composite sample from the zone in direct contact with the mat. Each sample was analysed in at least three analytical replicates.

Separate mats were allocated to the individual retrieval times. Mats from groups G1–G3 were retrieved after 60, 90 and 123 days. All analyses reported in the present paper, including ICP-OES, DMA, contact-angle measurements, roughness measurements and digital microscopy, refer exclusively to specimens collected on day 60. Materials retrieved on days 90 and 123 were retained for subsequent investigation and will be reported together with the replicated trial.

### 3.4. Elemental Analysis

Elemental concentrations were determined by inductively coupled plasma optical emission spectrometry (ICP-OES; Thermo Fisher Scientific, Waltham, MA, USA). Plant material was mineralised in accordance with PN-R-04014:1991 (chemical and agricultural analysis of plants—mineralisation of plant material for the determination of macro- and microelements). Soil and composting material were analysed following EN ISO 22036:2024 [36](determination of trace elements in soil, treated biowaste and compost by ICP-OES). Wool and exposed mats were digested with the same procedure as plant material, which was subsequently recognised as a possible cause of incomplete recovery of keratin-bound sulphur (Section 2.1). Each sample was analysed in at least three analytical replicates (*n* ≥ 3) and the mean is reported. Procedural blanks and certified reference materials were included in each digestion batch. The accuracy of the analytical procedure was evaluated using NIST SRM 1573a Tomato Leaves for plant-type samples and NIST SRM 2711a Montana II Soil for soil and compost samples. The certified reference materials were subjected to the same digestion and analytical procedures as the samples. Recoveries of the certified elements ranged from 91.2% to 108.6%, while the relative standard deviation for replicate determinations was below 8%. The limits of detection (LOD) and quantification (LOQ) were calculated as three and ten times, respectively, the standard deviation of ten procedural blanks divided by the slope of the corresponding calibration curve. Depending on the element, the method LOD and LOQ ranged from 0.01 to 2.5 mg kg^−1^ dry matter and from 0.03 to 8.3 mg kg^−1^ dry matter, respectively. Representative analytical wavelengths were 396.152 nm for Al, 188.980 nm for As, 249.773 nm for B, 317.933 nm for Ca, 214.439 nm for Cd, 267.716 nm for Cr, 324.754 nm for Cu, 259.940 nm for Fe, 766.490 nm for K, 285.213 nm for Mg, 257.610 nm for Mn, 231.604 nm for Ni, 220.353 nm for Pb, 196.026 nm for Se and 213.856 nm for Zn. Elements whose concentrations were below the LOQ were reported as <LOQ and excluded from relative comparisons. Results considered analytically implausible, as discussed in Section 2.1, are flagged in Table 1 and will be verified by ICP-MS. All concentrations are expressed in mg kg^−1^ dry matter.

### 3.5. Dynamic Mechanical Analysis

The viscoelastic behaviour of the mats was examined on a DMA 850 instrument (TA Instruments, New Castle, DE, USA) following the general principles of ISO 6721-1 [37]. Temperature sweeps were performed from −60 to 100 °C at 5 °C min^−1^ and 10 Hz. Three rectangular specimens per condition with nominal dimensions of 30 × 8 × 2 mm (length × width × thickness), tested in film-tension mode with a clamp separation of 15 mm, were cut from one mat per condition and tested in the same orientation; the reported parameters are means of the three specimens (*n* = 3). The modulus was calculated from the nominal external cross-section of the specimen. The undegraded material was tested at an oscillation amplitude of 15 µm and a force of 7 N; because composting substantially reduced the stiffness, the amplitude and force were decreased to 10 µm and 3–5 N for the composted specimen to remain within the instrument response range. E′, E″ and tan δ = E″/E′ were recorded using TRIOS software (version 5.8; TA Instruments, New Castle, DE, USA). E′ and E″ were evaluated at −30 and 50 °C, representing the limits of the anticipated service-temperature interval. The temperatures of the E″ and tan δ maxima were determined from the full sweeps, and the onset of the E′ decrease was defined as the temperature at which E′ fell by 10% relative to its maximum in the 40–75 °C interval. Storage-modulus retention was calculated as E′_composted_/E′_undegraded_ × 100%.

### 3.6. Water Contact Angle and Surface Roughness

Static water contact angles were measured before and after composting by the sessile-drop method with reference to ISO 19403-2 [38], using a DSA10 Mk2 drop-shape analyser (Krüss GmbH, Hamburg, Germany). Six measurable droplets were evaluated per condition, deposited at different positions on a single mat per condition; they are therefore technical replicates and not independent material replicates. Two additional droplets on the undegraded mat and four additional droplets on the composted mat were excluded because they were immediately absorbed by exposed fibres and did not form a stable profile suitable for contact-angle determination.

Because the mats had a porous, fibrous surface, droplets that were immediately absorbed by exposed fibres and could not be captured as a stable profile were excluded from the analysis. Surface roughness was measured with a Hommelwerke T1000 contact stylus profilometer (Jenoptik, Jena, Germany) equipped with a TK300 pick-up, using a traversing length of 4.80 mm and a Gaussian filter with a cut-off wavelength of 0.8 mm. Three profiles were recorded per condition, and the maximum height of the roughness profile, *R*_z_, was determined according to ISO 4287 [39]. The three profiles describe within-surface variability and do not constitute independent material replicates.

### 3.7. Digital Microscopy

The morphology of the exposed mats was examined with a VHX-5000 digital microscope (Keyence Corporation, Osaka, Japan) at 20×, 100× and 150× after removal of the samples from the soil/compost environment (60 days of exposure). Three-dimensional surface reconstructions were generated using the depth-composition and 3D Display functions, in which images acquired at successive focal planes were combined into an extended-depth-of-field image with the corresponding height information.

### 3.8. Statistical Analysis

ICP-OES results are reported as the mean of at least three analytical determinations per sample (*n* ≥ 3), and DMA parameters as the mean of three specimens per condition (*n* = 3). Because the experimental design comprised one plot per treatment, one composter and one mat per condition, these replicates characterise analytical and within-mat variability only; no inferential statistics were therefore applied to between-treatment differences, which are reported descriptively as relative differences. Contact-angle and *R*_z_ data are reported as mean ± standard deviation; Welch’s *t*-test, the non-parametric Mann–Whitney U test (α = 0.05), Hedges’ g and 95% confidence intervals of the mean differences were calculated to describe the separation between the undegraded and composted mat, with the explicit reservation that droplets and profiles obtained on one mat per condition are technical replicates and the resulting *p*-values do not test a material-level treatment effect.

## 4. Conclusions

Porous wool–PLA biocomposite mats were manufactured by carding washed sheep wool and bonding it with pneumatically sprayed PLA hot-melt, a solvent-free process that requires no drying stage and preserves the open fibrous structure. In a preliminary, unreplicated single-season field trial, forage from the plot with buried mats contained 47.8% more S and 21.9% more K than forage from the untreated plot, with smaller increases in Mn and Cu, whereas Ca, Mg, B and Mo concentrations were lower; the sulphur difference persisted after normalisation to Al, whereas most of the potassium difference did not. The sulphur response is consistent with the release of cystine-derived sulphur from wool keratin, while the potassium response probably reflects indirect effects on soil moisture and nutrient mobilisation. Composting produced a nutrient-enriched composting material, although its composition was also affected by dry-matter loss and mineral contamination.

After composting, the mats retained only 17–23% of their initial storage modulus between −30 and 50 °C, the relaxation of the PLA phase shifted by 7–10 °C to higher temperatures, and the water contact angle decreased markedly without an appreciable change in stylus roughness. Microscopy revealed swelling, fissuring, cavity formation and fibre exposure concentrated at wool–PLA interfaces. These observations are consistent with the hypothesised coupled mechanism in which hydrolysis of PLA ester bonds removes the temporary cohesion of the mat and opens the fibrous network to keratinolytic degradation; direct degradation markers are still required to confirm it.

Wool–PLA mats therefore appear to be a promising circular route for the valorisation of low-grade wool as a temporary, degradable carrier of keratin-bound sulphur, with the reservation that no soil-fertility or fertilising effect has been demonstrated here. The agronomic observations remain preliminary because the field plots were not replicated and soil physicochemical properties, dry-matter yield and nutrient uptake were not measured. The ongoing work includes replicated, randomised field plots with wool-only and PLA-only controls, measurement of plant dry-matter yield and total nutrient uptake, soil pH, EC, organic matter, available S and mineral N, monitoring of sulphate release, keratinase activity and the microbial community, analysis of compost leachate, determination of PLA molar mass and crystallinity, respirometric biodegradation tests, assessment of PLA fragment persistence and of the sanitary status of the wool, and ICP-MS verification of the flagged elemental values.

## Figures and Tables

**Table 1:** Elemental composition (mg kg^−1^ dry matter) of the washed wool, control soil, forage, composting material and exposed mat determined by ICP-OES. Δ denotes the relative difference between forage from the plot with buried mats (G1) and the control forage (G0). Values are means of at least three analytical determinations per sample (*n* ≥ 3); one field plot per treatment.

Element	Washed Wool	Soil G0	Forage G0	Forage G1	Δ G1 vs. G0 (%)	Composting Material G2	Exposed Mat G3 (Day 60)
Macronutrients							
Ca	6612.40	47,024.00	23,704.04	14,015.00	−40.9	30,519.96	3323.86
K	352.01	5182.00	23,401.67	28,527.87	+21.9	3276.61	60,305.82 ^†^
Mg	714.33	10,806.00	3035.83	2646.72	−12.8	5021.29	77.37
P	213.60	370.30	5454.46	4859.62	−10.9	6396.60	783.45
S	5289.30	334.10	2027.83	2996.77	+47.8	3379.32	32,401.26
Micronutrients							
B	3.03	2.43	58.19	30.17	−48.2	46.07	11.83
Cu	7.65	22.19	10.92	11.85	+8.5	16.55	9.55
Fe	736.50	14,070.00	522.76	533.91	+2.1	2862.41	616.69
Mn	14.70	520.10	68.60	79.21	+15.5	181.41	46.16
Mo	0.18	0.43	7.23	4.00	−44.7	5.80	1.95
Ni	2.31	21.55	23.14	22.20	−4.1	23.69	22.13
Zn	76.85	47.84	56.57	57.61	+1.8	110.10	145.63
Beneficial and other elements							
Na	589.20	287.70	122.31	113.74	−7.0	263.59	3753.15 ^†^
Se	0.22	2.81	0.10	0.02	n.r.	0.04	0.78
Si	251.80	—	—	—	—	—	—
Ag	0.02	0.14	0.40	0.31	−22.5	0.29	0.28
Al	406.32	18,793.00	372.62	436.06	+17.0	4218.00	449.05
As	187.34 ^†^	—	—	—	—	—	—
Ba	12.87	144.10	32.81	29.32	−10.6	68.37	7.88
Be	2.82 ^†^	—	—	—	—	—	—
Bi	14.65 ^†^	—	—	—	—	—	—
Cd	—	0.35	0.75	0.43	−42.7	0.79	0.23
Co	0.21	8.81	0.25	0.23	−8.0	1.23	0.49
Cr	8.31	26.99	40.18	36.32	−9.6	40.41	37.25
Ga	117.20 ^†^	—	—	—	—	—	—
In	0.04	—	—	—	—	—	—
Li	0.15	29.63	0.30	0.32	+6.7	2.74	0.54
Pb	2.10	9.59	1.21	1.10	−9.1	10.27	1.04
Sr	13.26	207.70	55.19	27.51	−50.2	69.15	6.75

— not determined. Values are means of *n* ≥ 3 analytical determinations per sample; one field plot per treatment, one composter and one mat per condition, so that no between-group dispersion is available. ^†^ Values flagged as analytically unreliable (As, Be, Bi and Ga in wool, determined only in this matrix and not confirmed by an independent technique; K and Na in the G3 mat, see text); they are shown for completeness only and are not used for interpretation. Se concentrations in forage were close to the limit of quantification and their relative difference is therefore not reported (n.r.).

**Table 2:** DMA parameters of the wool–PLA biocomposite mat before and after composting (10 Hz).

Parameter	Undegraded	Composted	Change
E′ at −30 °C (MPa)	2645	445	−83%
E′ at 25 °C (MPa)	≈2160	≈420	−81%
E′ at 50 °C (MPa)	2107	489	−77%
E′ (50 °C) − E′ (−30 °C) (MPa)	−538	+44	—
E″ at −30 °C (MPa)	217	28	−87%
E″ at 50 °C (MPa)	149	41	−72%
tan δ at −30 °C	0.082	0.063	—
tan δ at 50 °C	0.071	0.084	—
T_g_ (°C)	57.2	66.1	+8.9 °C
E″ maximum (°C)	≈65	≈75	+10 °C
tan δ maximum (°C)	≈73	≈80	+7 °C
Onset of E′ decrease (°C)	≈62	≈72	+10 °C

tan δ at −30 and 50 °C was calculated as E″/E′. Values at 25 °C and transition descriptors obtained from the full curves are approximate. Values are means of three specimens per condition (*n* = 3) cut from one mat per condition; because they describe within-mat variability only, the changes were not subjected to inferential statistics.

**Table 3:** Water contact angle (θ) and maximum roughness height (*R*_z_) of the wool–PLA mats before and after composting.

Parameter	Undegraded	Composted	Difference (95% CI)	*p* (Welch/M–W)	Hedges’ g
θ (°), *n* = 6	72.3 ± 10.0	55.7 ± 12.1	−16.5 (−30.8; −2.2)	0.028/0.015	−1.38
*R*_z_ (µm), *n* = 3	8.8 ± 4.7	12.0 ± 6.7	+3.2 (−10.6; +16.9)	0.54/1.00	0.44

Values are mean ± SD. Individual values: θ, undegraded: 85.5, 76.1, 71.4, 73.2, 72.5 and 54.8°; composted: 71.7, 69.2, 50.4, 53.8, 46.9 and 42.3°. *R*_z_, undegraded: 3.45, 10.94 and 12.12 µm; composted: 5.96, 19.23 and 10.80 µm. M–W, Mann–Whitney U test; CI, confidence interval. All droplets and profiles were obtained on one mat per condition and are technical replicates; the statistics describe within-mat differences only.

## Data Availability

The original contributions presented in this study are included in the article and Supplementary Materials. Further inquiries can be directed to the corresponding author.

## Supplementary Materials

The following supporting information can be downloaded at: https://www.mdpi.com/article/10.3390/molecules31203777/s1, Figure S1: Temperature dependence of the storage modulus (E′) and loss modulus (E″) of the wool–PLA mats before degradation and after composting; the vertical lines indicate the service-temperature range from −30 to 50 °C.

## Abbreviations

The following abbreviations are used in this manuscript:

CI	Confidence interval
DM	Dry matter
DMA	Dynamic mechanical analysis
DSC	Differential scanning calorimetry
E′	Storage modulus
E″	Loss modulus
ICP-OES	Inductively coupled plasma optical emission spectrometry
M–W	Mann–Whitney U test
PLA	Poly(lactic acid)
*R*_z_	Maximum height of the roughness profile
T_g_	Glass-transition temperature
