# Supplementary material for: Wool–Poly(lactic acid) Biocomposite Mats as Degradable Sulphur Carriers: Effects on Forage Elemental Composition and Material Degradation
Source: Molecules. 2026 Oct 21;31(20):3777. doi: 10.3390/molecules31203777 (PMC13707434; doi:10.3390/molecules31203777)
Supplement: Supplementary file 1 [file molecules-31-03777-s001.zip › molecules-4621544-supplementary.pdf]

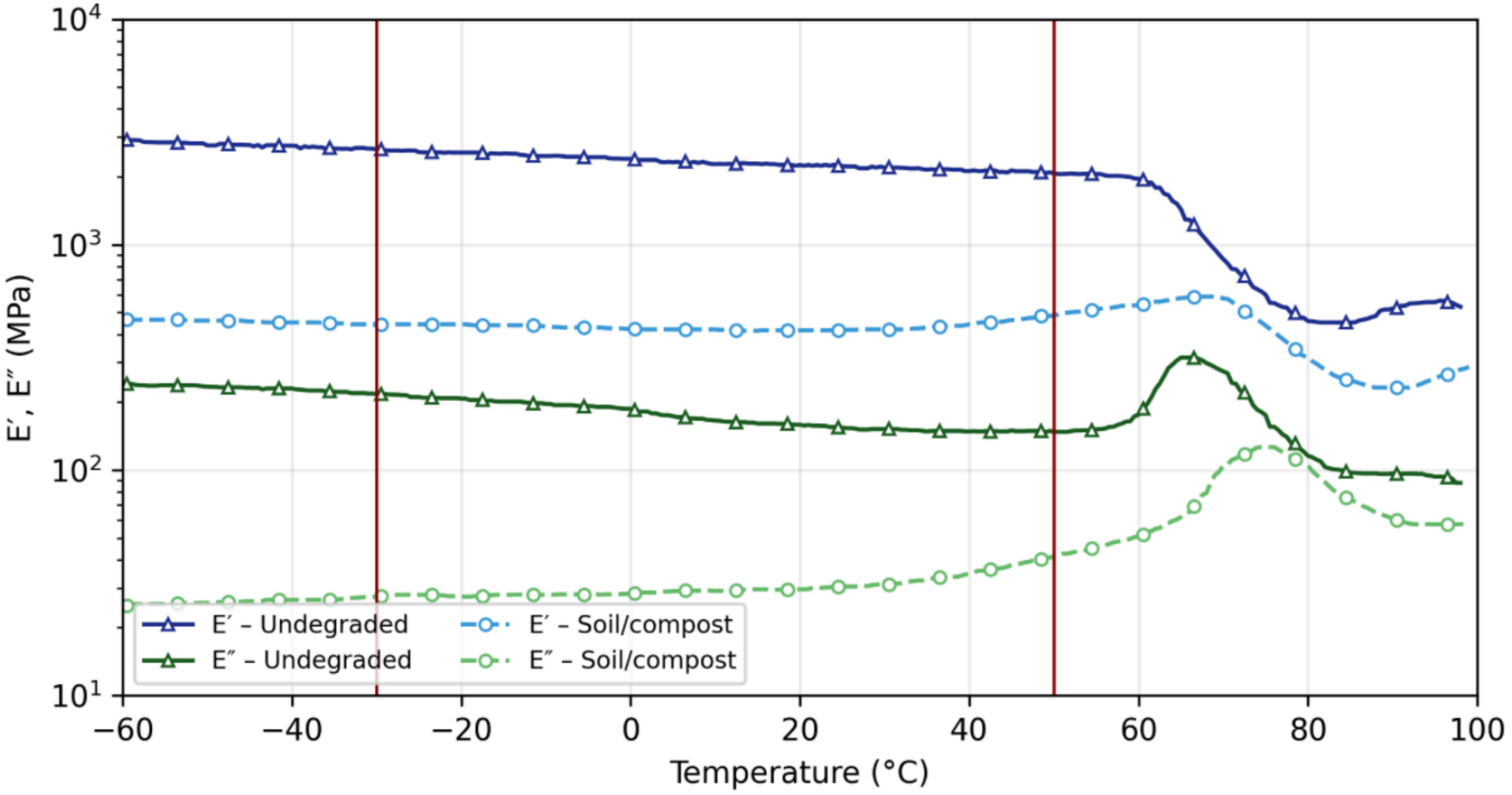

Figure S1. Temperature dependence of the storage modulus (*E*′) and loss modulus (*E*″) obtained by dynamic mechanical analysis (DMA) for undegraded specimens and specimens after soil/compost exposure. Solid lines with triangular markers denote undegraded specimens, whereas dashed lines with circular markers denote specimens after soil/compost exposure. Red vertical lines mark −30 and 50 °C.
